# Effect of information provision on parental intention toward COVID-19 vaccination for children: a nationwide survey experiment

**DOI:** 10.1038/s41598-024-56116-z

**Published:** 2024-03-04

**Authors:** Hyunju Lee, Jung Hyun Park, Shinkyeong Kim, Sujin Seo, Minjung Lee, Myoungsoon You, Eun Hwa Choi, Geun-Yong Kwon, Jee Yeon Shin, Min-Ah Lee, Mi Jin Jeong, Young June Choe, Syngjoo Choi

**Affiliations:** 1 Department of Paediatrics, Seoul National University Bundang Hospital, Seongnam-Si, Korea; 2 Department of Paediatrics, Seoul National University College of Medicine, Seoul, Korea; 3 Department of Economics. College of Social Sciences, Seoul National University, Seoul, Korea; 4 Department of Public Health Sciences, Graduate School of Public Health, Seoul National University, Seoul, Korea; 5 Yale School of Nursing, Yale University, New Haven, CT USA; 6 Division of Immunization, Korea Disease Control and Prevention Agency, Cheongju-si, Korea; 7 Korea Disease Control and Prevention Agency COVID-19 Vaccination Task Force, Cheongju-si, Korea; 8 Department of Paediatrics, Korea University Anam Hospital, Seoul, Korea

**Keywords:** Epidemiology, Paediatric research

## Abstract

The reluctance of parents to vaccinate their children against COVID-19 was prevalent particularly when uncertainty over vaccination outcomes prevailed. We conducted a nationwide randomized online survey experiment to assess the effect of information provision on parental intention for COVID-19 vaccination before the government started vaccination for children in South Korea. Parents of elementary school children were provided with either no information (Control), information on vaccine profile (vaccine informed group; VI), or COVID-19 (disease informed group; DI). Among 359,110 participants, parental intention for vaccination of children was significantly higher in both VI and DI groups compared with the Control group. In terms of effect size, information on COVID-19 vaccine increased likelihood to vaccinate by 1620 per 100,000 parents and reduced vaccine hesitancy by 1340 per 100,000 parents. Consistently with the positive effect on vaccination intention, both VI and DI interventions increased participants’ perceptions on vaccination benefits being higher than its risks and vaccination risks being lower than health risks of COVID-19 infection, and self-reported trust in COVID-19 information. Our results lend strong support to the claim that the provision of targeted, tailored information on COVID-19 vaccine and infection increases parental intention to vaccinate children and reduces vaccine hesitancy.

## Introduction

Vaccination is among one of the most effective public healthcare measures in reducing the burden of infectious diseases and associated mortality, especially in children^1^. The development of coronavirus disease 2019 (COVID-19) vaccine has made a significant impact in the epidemiology of the unprecedented severe acute respiratory syndrome coronavirus 2 (SARS-CoV-2) pandemic^2^. Despite this scientific breakthrough, vaccine uptake and increasing coverage has faced challenges in many populations^3^.

Vaccine development is a long, complex process in normal situations. The average vaccine requires a development timeline of 10–15 years from preclinical phase and market entry probability is 6%^4^. Clinical trials are conducted sequentially and approval is based on evidence that the vaccine is effective for its intended use, and the benefits of the vaccine outweigh the risk of adverse event^5^. However, the COVID-19 pandemic has expedited the development of COVID-19 vaccines in an unprecedented speed particularly with the help of the new platform of mRNA vaccines^6^. At the same time, this has brought on difficulties in obtaining public understanding and trust^7^.

Vaccine hesitancy, defined as a delay in acceptance or refusal of vaccines despite availability, is a major obstacle for vaccine uptake^8^. Multiple factors can potentially influence a parent’s decision to vaccinate themselves or their child^9,10^. Uncertainty for short- and long-term safety profiles or effectiveness of vaccines are the most commonly reported factors which contribute to vaccine hesitancy^7^. In this sense, COVID-19 vaccines are distinguished from vaccines developed from regular processes, especially vaccines provided in childhood which have been used widely for long periods of time^11^.

Decisions on vaccination in children differ from other populations. Decisions are largely dependent on caregivers’ opinions, and it is important to adopt a patient-centred approach. Clear, concise, evidence-based messages and efficient message delivery methods are crucial for informed-decision-making^12^. Strategies on risk communication is essential for developing appropriate vaccine policies for the target population^13^. At the time of our study, parents faced a unique and difficult situation in which they were required to make timely decisions for a newly developed mRNA vaccine amid the COVID-19 pandemic. Studies have demonstrated significant parental vaccine hesitancy for their children^14^. Relatively low vaccination uptake in children 5–11 years of age further reflects the challenges in this population^15^.

Although importance of informed decisions has been emphasized, there is still limited understanding on the actual effect of exposure to information on parental decision-making. Therefore, this study was performed to test the hypothesis that scientific-based vaccine-related information (safety and effectiveness) and infection-related information (incidence, severity and transmission) influence parents' decisions to vaccinate their children, especially when uncertainty about short- and long-term safety profiles or effectiveness of vaccines was prevailing. In this study, we conducted a large-scale, nationwide experimental survey to assess the effect of information provision on parental intention for COVID-19 vaccination and propose effective measures for public health communication in vaccine policies in children.

## Methods

### Study design

A nationwide survey was conducted among parents of elementary school children. The survey was performed as a parallel-group, randomized controlled trial. Intervention and data collection were carried out online. Participants in each group were randomly assigned a questionnaire which included information on COVID-19 infection or vaccination. Randomization was performed at the school level from the entire list of elementary schools throughout in the Republic of Korea with information on latitude and longitude of schools (refer to Sect. 1 of Supplementary Materials). Because of privacy concerns, we only collected information on children’s school district and region.

The study was conducted, using an online platform, between February 7 and February 10, 2022, before the Korean government started to vaccinate children 5–11 years of age at the end of March, 2022. At the time of the study, evidence on vaccine safety and effectiveness for this age group was limited. Thus, participants were likely to respond to the survey while vaccine uncertainty prevailed. The survey was sent out to parents of elementary school children in web-based school notices, commonly used for announcements at all schools. Responses from parents were collected voluntarily and anonymously and no personal identification data were obtained. Parents were informed to specifically address only one child in households with more than two children. The survey was performed in collaboration with the Ministry of Education and research fund provided by the Korea Disease Control and Prevention Agency (KDCA).

### Survey experiment

**Figure 1:**
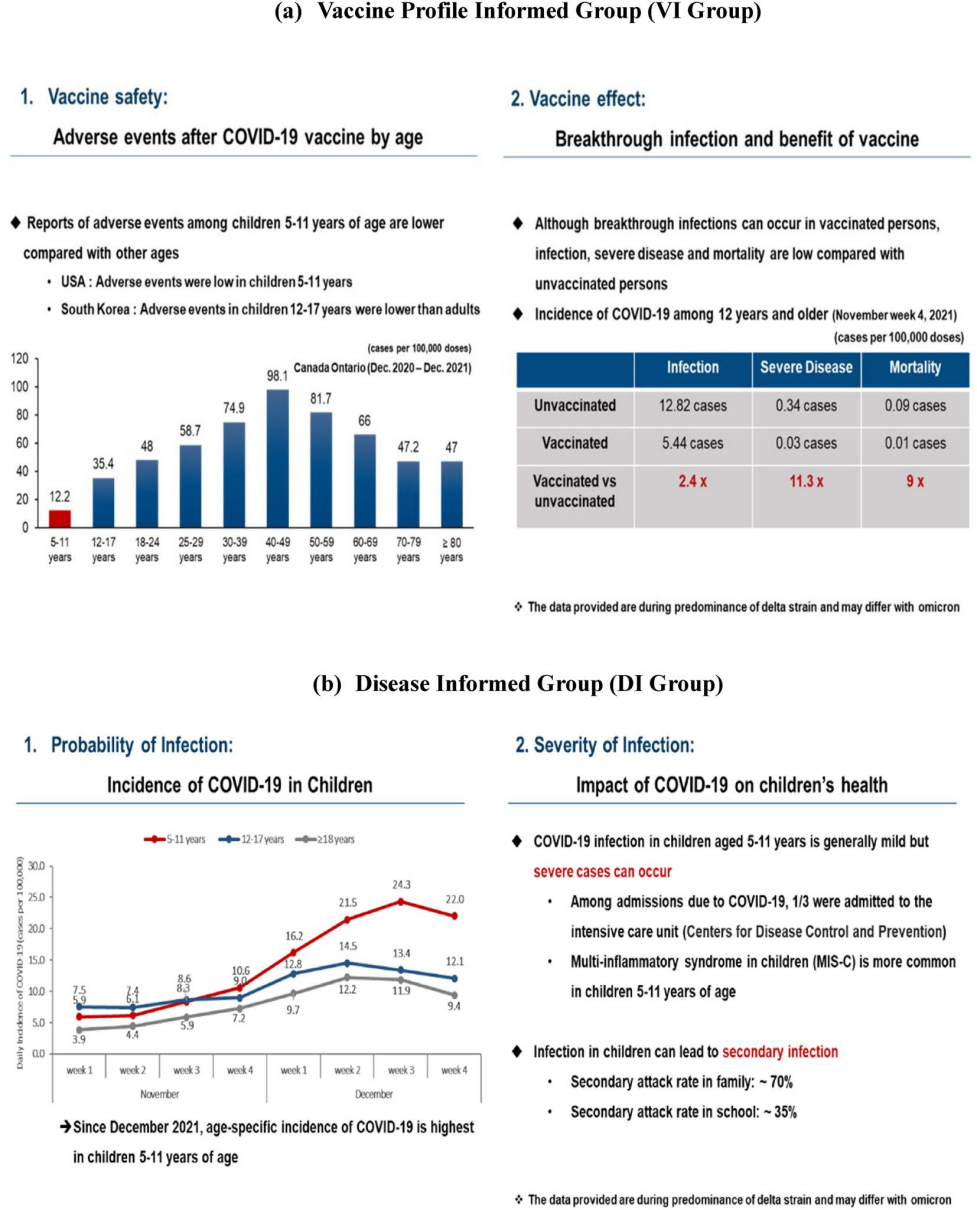
Intervention for parental survey. Information intervention for Vaccine Profile Informed Group (VI group) showed differences in adverse events by age groups and vaccine effect compared with unvaccinated subjects. Information intervention for Disease Informed Group (DI group) showed incidences of COVID-19 according to age groups and impact of COVID-19 on children’s health.

To examine the impact of information provided on acceptance for COVID-19 vaccination in children, parents were randomly assigned online to one of three different surveys. Different information was provided to parents as intervention. All three questionnaires consisted of the same questions except for information interventions. The first section of the survey consisted of questions on demographic characteristics of the child such as school grade, year of birth, sex, school district, self-reported child health status, and recent vaccinations. The second section included questions of perceptions on COVID-19 and the vaccine, parental COVID-19 vaccination status, perception on risk of child’s infection regarding possibility and severity and degree of confidence on information for children’s COVID-19 vaccination. After these questions, according to random assignment, participants were provided with either no information (Control Group), information on vaccine effectiveness and safety (Vaccine Profile Informed Group; henceforth called VI Group), or information on age-related incidence and impact of COVID-19 infection on children’s health (Disease Informed Group; henceforth called DI Group). Information provided was developed by a multi-disciplinary team consisted of pediatric infectious disease specialists, experts in public health communication and behavioural economists. VI Group was provided with age-dependent safety data reported in Canada, the US and KDCA and data reported by the KDCA of vaccine effectiveness for infection itself, critical infection and mortality among subjects 12 years of age and older^16–20^. DI Group was provided with data reported by the KDCA on recent changes in age-related incidence after implementation of vaccination in subjects 12 years of age and older, clinical spectrum of COVID-19 in children 5–11 years of age reported by the Centers for Disease Control and Prevention (CDC) and transmission rates of children in family and school settings^21,22^. Information about the two treatments is included in Fig. 1.

After exposure to the information, the survey continued to ask parents about their intention to vaccinate their children, perception on vaccine safety, vaccine effectiveness, vaccine risk–benefit and comparison of infection risk vs. vaccine risk. A translated version of the survey is reported in Sect. 2 of Supplementary Materials.

### Outcome

The main outcome variable was parents’ intention to vaccinate their children. This question was asked right after exposure to the randomized information treatments. Respondents were asked to pick one response from the following 5 options: “willing to get my child vaccinated as soon as possible”, “willing to get my child vaccinated but I want to wait and see”, “not willing to get my child vaccinated but I want to wait and see”, “Not willing to get my child vaccinated at all”, and “Don’t know/not sure”. Parental intention to vaccinate their children was categorized into three different variables: the first is one for strong vaccination intention defined as a response with “willing to get my child vaccinated as soon as possible”, the second for strong or moderate vaccination intention including further “willing to get my child vaccinated but I want to wait and see”, and the third for vaccine hesitancy defined to include two negative responses– “not willing to get my child vaccinated but I want to wait and see”, “Not willing to get my child vaccinated at all”. We report the treatment effects of all three types of vaccination intentions.

Along with vaccination intention, we also examined the effects of information intervention on respondents’ perception on risk–benefit of COVID-19 vaccine, risk comparison of COVID-19 infection and vaccine, and self-reported trust in COVID-19 vaccine information. These perceptions and attitudes variables are considered determinant factors for parents’ vaccination intention. If information interventions affect parental intention to vaccinate their child, they are expected to affect these perceptions and attitudes variables in the same direction.

Because respondents take time to process information presented in the intervention groups, we also examine survey response time as a first-stage outcome (Table 7S in Supplementary Materials).

### Ethics statement

This study was in support of the Korea Disease Control and Prevention Agency and Ministry of Education to be utilized for public interest in developing vaccination policies for children and was exempted by the Institutional Board Review of Seoul National University Bundang Hospital oversight (IRB No. X-2203–743-901) with a waiver for informed consent. All methods in this study were carried out in accordance with the relevant guidelines and regulations of the Declaration of Helsinki.

### Statistical analysis

We used the following regression specification to estimate the causal effect of our information interventions:

$${Y}_{i}={\beta }_{0}+{\beta }_{1}{T}_{1}+{\beta }_{2}{T}_{2}+{\gamma X}_{i}+{\varepsilon }_{i}$$

where ${Y}_{i}$ denotes an outcome variable of interest for individual $i$. ${T}_{j}$ is a binary indicator of Treatment group $j$ for $j=\mathrm{1,2}$. ${X}_{i}$ is a control vector that includes individual i and their children’s characteristics including the number of children, province of residence, parent’s COVID-19 vaccination status, confidence on COVID-19 vaccine information, for a child of interest, gender, school grade, health condition, past 5-year status of vaccination, perceptions about chance of COVID-19 infection and health risk of COVID-19 infection. The coefficient ${\beta }_{j}$ captures the average treatment effect of Treatment group $j$ relative to the Control group. Robust standard errors with clustering at the region level (among 17 different regions of Republic of Korea) are reported, because each region has an independent, autonomous education authority and differs in terms of regional patterns in the spread of COVID-19 infection. Although random assignment was made at the school level, we did not collect information on children’s school due to privacy concerns and were unable to cluster at the school level.

Our statistical analysis of estimating average treatment effects proceeded in two steps. We conducted a regression analysis without any control variable in the first step. Next, we estimated the treatment effects by controlling the full set of covariates including demographic information and respondents’ perception about COVID-19 infection and vaccines. Because the treatment effects remain robust across different specifications, we report the average treatment effects with the full set of controls. The results across all specifications report in Table 6S in Supplementary Materials. The normalized difference of mean response rates between treatment group and control group (normalized DRR) to the mean response rate in the control group was also analysed. We also estimated heterogeneous treatment effects across subgroups of the sample with the full set of controls.

## Results

Information on the baseline characteristics of participants who completed the survey, from 6,323 elementary schools in the Republic of Korea are summarized in Table 1. The total number of participants in our study were 359,110, which covers 13.4% of students in the population^23^ (Comparison between total population and participants in survey is shown in Table 2S). Among subjects, 51.4% (184,648) of the children were male and school grades of children ranged from 17.1% (61,335) in 2nd grade to 21.6% (77,593) of 6th grade and 3.1% (11,251) were in 1st grade. Most children were reported to be healthy at the time of the study (318,370; 88.7%) and 88.1% (316,381) of children had experiences of vaccination in the past 5 years. Most parents reported that they got at least two shots of COVID-19 vaccine (317,209; 88.3%). Regarding parental perceptions related to COVID-19 infection, 23.6% (84,901) of parents perceived that the chances of their child getting COVID-19 infection are low, 45.5% (163,509) of parents had a perception that their child’s health risk from COVID-19 infection is severe. Among parents, 25.5% (91,629) reported a very low level of confidence on COVID-19 vaccine information that they accessed. There are no statistical differences with respect to these baseline variables among the participants across the three groups (refer to Table 3S in Supplementary Materials).

**Table 1:** Summary of participant characteristics.

	Variable	Total (*N* = 359,110)		Control Group (*N* = 113,450)		VI Group (*N* = 117,264)		DI Group (*N* = 128,396)	
Gender of a child	Male	184,648	(51.4)	58,342	(51.4)	60,463	(51.6)	65,843	(51.3)
	Female	174,462	(48.6)	55,108	(48.6)	56,801	(48.4)	62,553	(48.7)
School grade	Grade 1	11,251	(3.1)	3,598	(3.2)	3,562	(3.0)	4,091	(3.2)
	Grade 2	61,355	(17.1)	19,315	(17.0)	20,078	(17.1)	21,962	(17.1)
	Grade 3	62,106	(17.3)	19,482	(17.2)	20,410	(17.4)	22,214	(17.3)
	Grade 4	71,617	(19.9)	22,718	(20.0)	23,331	(19.9)	25,568	(19.9)
	Grade 5	75,188	(20.9)	23,917	(21.1)	24,461	(20.9)	26,810	(20.9)
	Grade 6	77,593	(21.6)	24,420	(21.5)	25,422	(21.7)	27,751	(21.6)
Number of children	1, 2 children	310,168	(86.4)	97,910	(86.3)	101,109	(86.2)	111,149	(86.6)
	3 + children	48,942	(13.6)	15,540	(13.7)	16,155	(13.8)	17,247	(13.4)
Region	Seoul, Incheon	84,253	(23.5)	26,261	(23.2)	25,161	(21.5)	32,831	(25.6)
	Gyeonggi	83,804	(23.3)	28,641	(25.3)	24,332	(20.8)	30,831	(24.0)
	Daejeon, Sejong, Chungcheong	34,801	(9.7)	10,941	(9.6)	12,108	(10.3)	11,752	(9.2)
	Gwangju, Jeolla	34,726	(9.7)	12,066	(10.6)	11,315	(9.7)	11,345	(8.8)
	Busan, Ulsan, Gyeongsangnam-do	67,560	(18.8)	19,187	(16.9)	25,461	(21.7)	22,912	(17.8)
	Daegu, Gyeongsangbuk-do	38,330	(10.7)	11,308	(10.0)	14,234	(12.1)	12,788	(10.0)
	Gangwon, Jeju	15,636	(4.4)	5,046	(4.5)	4,653	(4.0)	5,937	(4.6)
Health status of a child	Healthy	318,370	(88.7)	100,535	(88.6)	104,057	(88.7)	113,778	(88.6)
	Moderate	37,086	(10.3)	11,778	(10.4)	11,983	(10.2)	13,325	(10.4)
	Unhealthy	3,654	(1.0)	1,137	(1.0)	1,224	(1.0)	1,293	(1.0)
Past vaccination of a child	Yes	316,381	(88.1)	99,979	(88.1)	103,233	(88.0)	113,169	(88.1)
	No	20,617	(5.7)	6,439	(5.7)	6,724	(5.7)	7,454	(5.8)
	Unknown	22,112	(6.2)	7,032	(6.2)	7,307	(6.2)	7,773	(6.1)
Parent's COVID-19 vaccination	3rd shot	174,960	(48.7)	55,432	(48.7)	56,913	(48.5)	62,615	(48.8)
	2nd shot	142,249	(39.6)	44,796	(39.5)	46,650	(39.8)	50,803	(39.6)
	1st shot & none	41,901	(11.7)	13,222	(11.7)	13,701	(11.7)	14,978	(11.7)
Chance of COVID-19 infection (child)	Unlikely	84,901	(23.6)	26,845	(23.7)	27,797	(23.7)	30,259	(23.6)
	Neutral	205,222	(57.2)	64,550	(56.9)	67,209	(57.3)	73,463	(57.2)
	Likely	68,987	(19.2)	22,055	(19.4)	22,258	(19.0)	24,674	(19.2)
Health risk of COVID-19 infection (child)	Mild	50,799	(14.2)	15,894	(14.0)	16,516	(14.1)	18,389	(14.3)
	Moderate	144,802	(40.3)	45,462	(40.1)	47,430	(40.5)	51,910	(40.4)
	Severe	163,509	(45.5)	52,094	(45.9)	53,318	(45.5)	58,097	(45.3)
Confidence on COVID-19 vaccine information	Not at all	91,629	(25.5)	29,124	(25.7)	29,598	(25.2)	32,907	(25.6)
	Somewhat	217,151	(60.5)	68,595	(60.5)	71,213	(60.7)	77,343	(60.2)
	Completely	50,330	(14.0)	15,731	(13.9)	16,453	(14.0)	18,146	(14.1)

### Average treatment effects of information interventions

**Figure 2:**
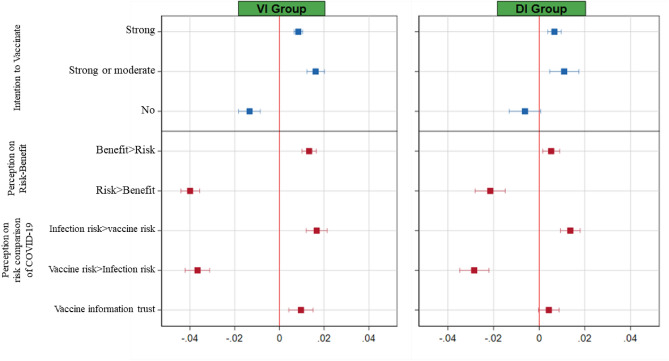
Average Treatment Effects on Parental Vaccination Intention for Children and Its Determinant Factors.

Figure 2 reports the average treatment effects of outcomes of interest and their 95 percent confidence intervals with controlling the full set of covariates: three different types of parental intentions to vaccinate their children—strong intention, strong or moderate intention, and vaccine hesitancy (no vaccination intention)—and factors determining the parental vaccination intention. Each outcome variable is defined to be binary. The corresponding regression tables are reported in Table 4-2S and Table 5S in Supplementary Materials.

Let us first present the treatment effects on parental vaccination intentions. Starting with parental strong intention to vaccinate their children, information on COVID-19 vaccine increased strong vaccination intention by 0.9%p and exposure to information of COVID-19 infection in children increased strong intention by 0.7%p relative to the Control Group. Given the average of strong vaccination intentions in the Control Group (6.5%), these treatment effects amounted to a 13% increase in the VI Group (*P* < 0.001) and 10% increase in the DI Group (*P* < 0.001). Turning to parental strong or moderate intention, being assigned to the VI Group or DI Group increased vaccination intention by 1.6%p or 1.1%p, respectively. Given the average of strong or moderate vaccination intentions in the Control Group (35.4%), effects of information provision amounted to a 5% increase in the VI Group (*P* < 0.001) and 3% increase in the DI Group (*P* < 0.005). Finally, information provision reduced vaccine hesitancy (no vaccination intention) by 1.3%p in the VI Group and 0.6%p in the DI Group. Effects of information provision on vaccine hesitancy amounted to a 2.2% decrease in the VI Group (*P* < 0.001) and 1% reduction in the DI Group (*P* = 0.074). In summary, the provision of evidence-based scientific information on COVID-19 vaccine and COVID-19 infection increased parental intention to vaccinate their children and reduced vaccine hesitancy. The impact of providing information on COVID-19 vaccine (the VI treatment) increased the likelihood of parents expressing strong or moderate intentions to vaccinate their children by 1620 per 100,000 parents and reduced the likelihood of parents expressing vaccine hesitancy by 1,340 per 100,000 parents in the population. Informing parents of evidence on COVID-19 infection (the DI treatment) also had significant effects, albeit to a less degree. Given negligible costs of communicating evidence-based scientific information on COVID-19 vaccine to parents, this kind of information interventions can be beneficial from the perspective of cost–benefit analysis.

We turn to the treatment effects on several parental perceptions about COVID-19 vaccine which can be considered determinants of vaccination intention. We find that the information effects on parental perceptions are in line with those on parental vaccination intention. With respect to perceptions about risk and benefit of COVID-19 vaccine, the VI intervention (resp. the DI intervention) increased the response rate of the benefit of COVID-19 vaccine being higher than its risk by 1.3%p (resp. 0.5%p) and decreased the response rate of the risk of COVID-19 vaccine being higher than its benefit by 4.0%p (resp. 2.2%p). Given that 11.9% of the participants in the Control Group reported higher benefit of getting their child vaccinated than risk and 51.7% in the Control group reported the opposite, the VI intervention leads to an 11.2% increase in the positive perception about risk–benefit of COVID-19 vaccine and a 7.7% decrease in the negative perception (*P* < 0.001). Similarly, but to a lesser degree, the DI intervention leads to a 4.4% increase in the positive perception (*P* < 0.01) and a 4.2% reduction in the negative perception (*P* < 0.001) about the risk–benefit comparison of COVID-19 vaccine. Hence, the information interventions increased the positive perception and reduced the negative perception on risk–benefit comparison of COVID-19 vaccine, in accordance with the treatment effects on parental vaccination intentions.

Regarding the parental perception about comparing risks from COVID-19 infection and COVID-19 vaccination, 14.9% of participants in the Control group reported health risk from COVID-19 infection higher than that from taking COVID-19 vaccine and 52.1% in the Control Group reported the opposite response that health risk from taking COVID-19 vaccine is higher than that from COVID-19 infection. The VI intervention increased the perception of COVID-19 infection risk being higher than COVID-19 vaccine risk by 1.7%p, amounting to an 11.2% increase relative to the mean response rate in the Control Group (*P* < 0.001), and reduced the opposite perception by 3.7%p, amounting to a 7% decrease (*P* < 0.001). Similarly, the DI intervention increased the perception of COVID-19 infection risk being higher than COVID-19 vaccine risk by 1.4%p, amounting to a 9.1% increase (*P* < 0.001), and reduced the opposite perception by 2.9%p, amounting to a 5.5% decrease (*P* < 0.001). Thus, information interventions increased the positive perception about risk comparisons and reduced the negative perception. These findings on the parental perception on risk comparisons are again in line with the treatment effects on vaccination intentions.

Finally, the effects of the information interventions on self-reported trust in COVID-19 vaccine information are also consistent with those on vaccination intention: the VI intervention increased respondents’ trust by 1%p and the DI intervention did so by 0.4%p. Given that 20.3% of the participants in the Control Group reported that they trust information about COVID-19 vaccine, these intervention effects amount to a 4.7% increase in parental trust in COVID-19 vaccine information in the VI Group (*P* < 0.005) and a 2.1% increase (however not statistically significant) in the DI Group. Hence, providing evidence-based information in our setup increased parental trust in COVID-19 vaccine information, which can explain partly why the information interventions affect parental vaccination intention.

### Heterogeneous treatment effects on parents’ vaccination intention

**Figure 3:**
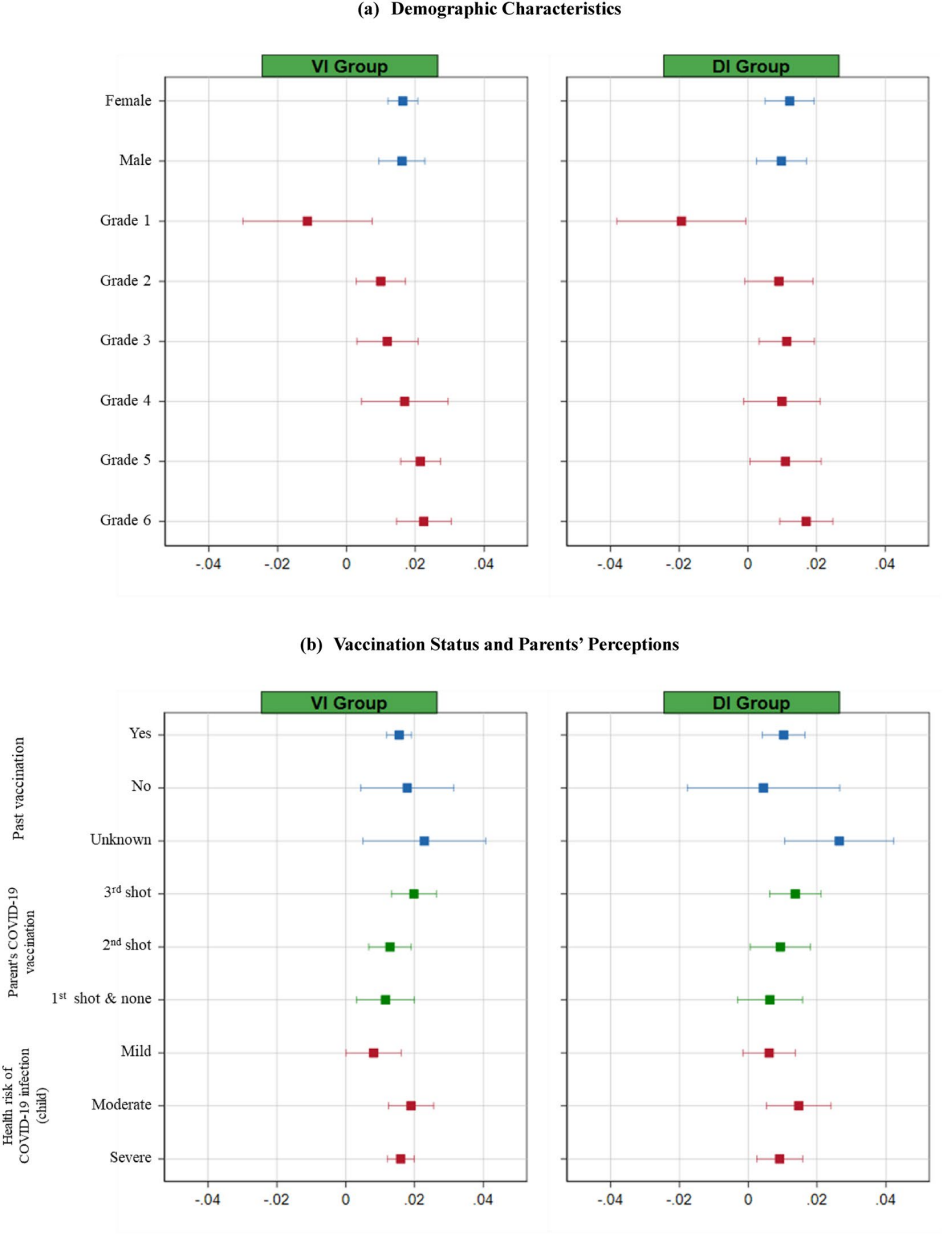
Heterogeneous Treatment Effects on Parental Vaccination Intention for Their Children.

Figure 3a displays the heterogeneous treatment effects across subgroups with gender and school grade of a child and Fig. 3b across subgroups regarding a child’s status of vaccination in the past 5 years, parents’ status of COVID-19 vaccination, and parental perceptions on a child’s health risk of COVID-19 infection. The VI intervention showed decreased parental intention to vaccinate their child by 1.1%p among children in the 1st grade (*P* > 0.05), and then increased it by 1%p in 2nd grade, 1.2%p in 3rd grade, 1.7%p in th grade, 2.2%p in 5th grade, and 2.3%p in children in 6th grade. We found a similar pattern for the DI intervention. The parental intention was significantly lower in 1st grade compared with other grades in both VI and DI intervention, respectively. This attributes to the lower perceived risk for COVID-19 infection and impact of infection on child’s health compared with children in other grades (*P* < 0.001).

Parents who received the third shot of COVID-19 vaccine were 2.0%p and 1.4%p more responsive to each of the VI and DI interventions than those who received either the 2nd shot or first shot or none. The VI intervention effect on vaccination intention was 1.6% larger when respondents perceived severe health risks of COVID-19 infection for a child compared with perception on mild or moderate health risks. A similar heterogeneous effect for the DI intervention effect was observed for this variable. The corresponding regression table is reported in Table 6S in Supplementary Materials.

## Discussion

In this study, we found that exposure to evidence-based scientific information on COVID-19 vaccines and COVID-19 infection significantly increased parental intent and reduced hesitancy for COVID-19 vaccination for children. Among information interventions, information on age-specific incidence of adverse events and vaccine effectiveness had a higher impact on intention for vaccination compared with information on COVID-19 infection. In addition to intention to vaccinate children, the information provided to the intervention groups also enhanced positive perception and decreased negative perception on risk–benefit of COVID-19 vaccine, risk comparison of infection and COVID-19 vaccine, and self-reported trust to COVID-19 information.

When estimating the actual impact of the interventions, information on COVID-19 vaccines increased strong to moderate vaccination intention by 1.6%p and reduced vaccine hesitancy by 1.3%p relative to the control group, respectively. In other words, COVID-19 vaccine profile information increased the likelihood of parents to vaccinate their children by 1620 per 100,000 parents and reduced the likelihood of parents expressing vaccine hesitancy by 1,340 per 100,000 parents in the population. The methodology used in this study may also be considered beneficial from a cost–benefit perspective. With negligible costs, information interventions of evidence-based scientific information on COVID-19 and COVID-19 vaccines to parents resulted in a substantial increase in parental intentions for COVID-19 vaccination for their children.

Intervention studies to increase the acceptance of COVID-19 vaccination via information providing messages, behavioural nudges, virtual interventions, and videos have been reported^24–26^. Moreover, decision aids to support vaccination decision-making have been developed^27,28^. However, none of the previously reported interventions were targeted to parents with children and this study is distinguished as it was a nationwide study. Compared with previous studies, although the mean exposure time for information provided in this study was relatively short, the impact was significant. In this study, brief exposure to information on COVID-19 vaccines or COVID-19 infection enhanced not only perception on risk–benefit of COVID-19 vaccine and comparison of risk of vaccination and infection but also self-reported trust of COVID-19 vaccine. These aspects contribute to parental decisions for vaccination in their children on in a comprehensive manner. In addition, the tailored messages provided may have effectively affected the perception and motivation of parents. Parents are placed in an overwhelming “infodemic”, and tailored, targeted health communication may serve as a strategy to enhance processing health information and lead to changes in perception and behavior^29,30^.

In the heterogeneous treatment effects across subgroups in this study, we found that although there were no noticeable differences across the gender of the child, parental intentions for their child’s vaccination increased with increase in school grade of child, parents’ status of COVID-19 vaccination, and self-perceived health risks for COVID-19 infection in children. Considering information interventions provided in this study were tailored and targeted to parents of elementary school children, further specific messages may be necessary for children in lower grades. Studies on effective tailored, targeted messages for children of younger ages should be considered.

There are limitations of the study. First, it was conducted online, and thus the participants may not be representative of the population, because participation may depend on access to the internet and familiarity with online studies. Second, as the responders accounted for 13.4% of the targeted population, there may be a potential selection bias in the responders. However, the total number of participants were 359,110, which is a substantially large number, and among the highest to the authors’ knowledge regarding the subject of COVID-19 vaccine for children. When comparing the distribution of the sex, grade, districts of the total targeted population with the survey population, there was no difference except for low participation in the 1st graders. Also, this study randomly assigned participants to control and experimental groups, and as evident from the balance checks results, there were no differences between observed variables among the control group and each experimental group. Therefore, it can be inferred that the response and selection biases have been relatively well controlled. Third, the participation of parents among 1st graders were lower compared with other grades which may be related to the survey being performed before the beginning of the new grade year. Fourth, the decision to design a relatively short questionnaire was made with the primary goal of ensuring participant privacy and encouraging survey completion, and this design may have resulted in the omission of certain crucial covariates, specifically the socioeconomic levels of caregivers and information regarding previous adverse effects after vaccination. However, participants were randomly assigned and confirmed through balance checks, therefore the influence of these variables on the treatment effect has been controlled. Fourth, as this study was performed before vaccines were available for children 5–11 years of age, we were not able to explore whether the messages provided lead to actual vaccination.

Regardless of these limitations, in this nationwide survey of parents, we found that tailored, targeted messages based on evidence based scientific information effectively promoted parental intentions for their child’s COVID-19 vaccination, enhanced positive perception and reduced negative perception of risk–benefit of COVID-19 vaccine, risk comparison of infection and COVID-19 vaccine and self-reported trust in COVID-19 information. Among information interventions, information on vaccine such as safety and effectiveness had a greater impact compared with information on COVID-19 infection. This study provides evidence on effective methods for public health communication for vaccine policies in children.

### Supplementary Information

Supplementary Information.

## Data Availability

The study protocol and data files (containing the commands used) can be made available upon request. However, the de-identified individual participant data that underlie the results reported in this article are the property of the Korea Disease Control and Prevention Agency and cannot be made available. Please contact the corresponding author for further information.
