# Supplementary material for: Effect of information provision on parental intention toward COVID-19 vaccination for children: a nationwide survey experiment
Source: Sci Rep. 2024 Mar 4;14:5354. doi: 10.1038/s41598-024-56116-z (PMC10912657; doi:10.1038/s41598-024-56116-z)
Supplement: Supplementary file 1 — Supplementary Information. [file 41598_2024_56116_MOESM1_ESM.docx]

Supplementary Materials for

**The effect of information provision on parental intention toward COVID-19 vaccination for children:**

**A nationwide survey experiment**

1. **Data and random assignment of schools**

The data of elementary schools in the Republic of Korea was obtained from Open Government Data Portal (<https://www.data.go.kr/data/15049381/fileData.do>) in which various data held by the government are opened in accordance with the Act on Promotion of the Provision and Use of Public Data (Open Government Data Act).

It contains information on 6,323 elementary schools in Korea, and the number and ratio of elementary schools belonging to each regional office of education are shown in the table below.

| **Region** | **Freq.** | **Percent (%)** |
| --- | --- | --- |
| Seoul | 604 | 9.55 |
| Busan | 304 | 4.81 |
| Daegu | 232 | 3.67 |
| Incheon | 270 | 4.27 |
| Gwangju | 154 | 2.44 |
| Daejeon | 150 | 2.37 |
| Ulsan | 122 | 1.93 |
| Sejong | 51 | 0.81 |
| Gyeonggi | 1,338 | 21.16 |
| Gangwon | 374 | 5.91 |
| Chungcheongbuk-do | 268 | 4.24 |
| Chungcheongnam-do | 422 | 6.67 |
| Jeollabuk-do | 422 | 6.67 |
| Jeollanam-do | 462 | 7.31 |
| Gyeongsangbuk-do | 507 | 8.02 |
| Gyeongsangnam-do | 524 | 8.29 |
| Jeju | 119 | 1.88 |
| Total | 6,323 | 100 |

The data includes information about longitude, latitude, address of each elementary school, city and provincial office of education code, education support office code, and establishment date. For school-level random assignment, we used the information of latitude and longitude of school location after applying stratification using the Office of Education support code.

The following maps in Figure 1S show the results of random assignment in Seoul and in the entire area of South Korea. Group 1 represents the DI (Disease Informed) group, Group 2 the VI (Vaccine Profile Informed) group, and Group 3 the Control group.

**Figure 1S.** Maps of random assignment in Seoul (A) and in the entire area of South Korea (B)

1. Random assignment in Seoul


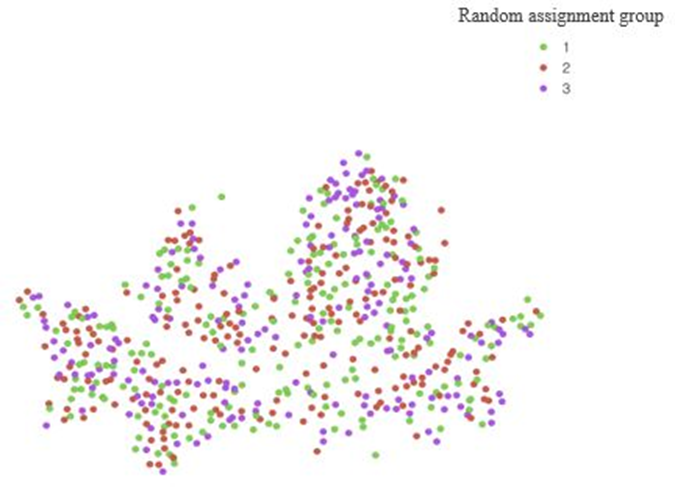


1. Random assignment in Korea


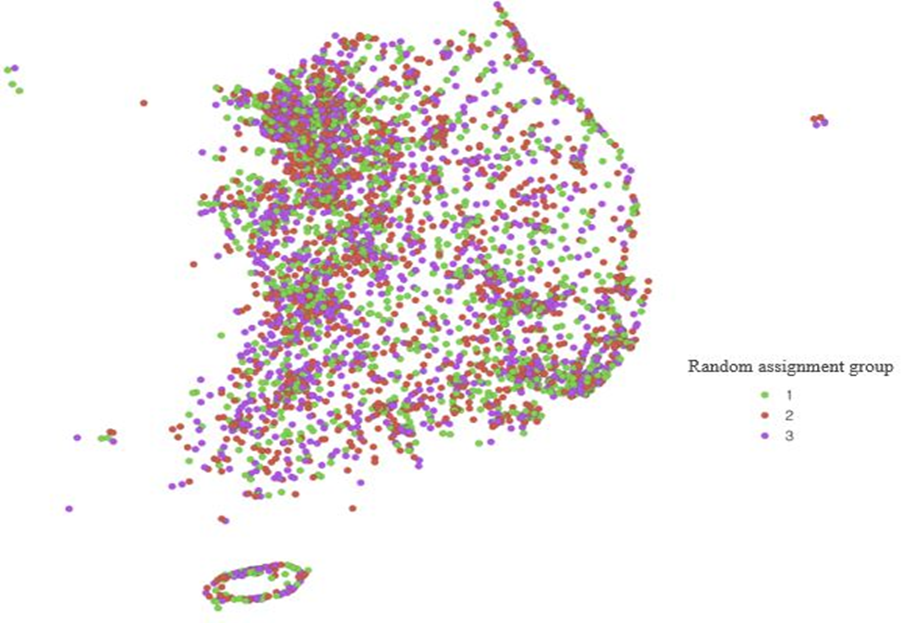


Table 1S reports the balance check of school assignment across the three groups using the information of latitude and longitude of school location. School assignment was well balanced because there is no significant difference in these location variables.

Table 1S. Balance checks using latitude and longitude information

| Variables | DI group | VI group | Control group |  |
| --- | --- | --- | --- | --- |
|  | Group 1  (Std.errors) | Group 2  (Std.errors) | Group 3  (Std.errors) | F-value  (p-value) |
| Latitude | 36.44 | 36.41 | 36.45 | 0.849 |
|  | (0.0371) | (0.0985) | (0.0319) | (0.429) |
| Longitude | 127.52 | 127.5 | 127.51 | 0.411 |
|  | (0.0290) | (0.0784) | (0.0285) | (0.664) |

1. **Sample Survey Questionnaire in the Vaccine profile informed group (VI group)**

This survey questionnaire is an English translation version of the questionnaire provided to the VI group. The control group and the DI group used the same questionnaire except for the figural information presented between Q11 and Q12 as information intervention. The links for original Korean version of the survey questionnaires are provided at the end of the sample survey questionnaire.

.

**1. How many children do you have in total?**

1) 1 child 2) 2 children 3) 3 children 4) 4 or more children

**<If you have more than two children, please think of one child in elementary school 1st to 6th, and answer all the questions below.>**

**2. Please select your child's birth year.**

1) 2015 2) 2014 3) 2013 4) 2012 5) 2011 6) 2010 7) 2009 8) others

**3. What grade will your child enter in March this year?**

1) Grade1 2) Grade2 3) Grade3 4) Grade4 5) Grade5 6) Grade6

**4. What is your child's gender?**

1) Female 2) Male

**5. In which of the following areas is your child's school?**

1) Seoul 2) Busan 3) Daegu 4) Incheon 5) Gwangju 6) Daejeon 7) Ulsan

8) Sejong 9) Gyeonggi 10) Gangwon 11) Chungcheongbuk-do

12) Chungcheongnam-do 13) Jeollabuk-do 14) Jeollanam-do

15) Gyeongsangbuk-do 16) Gyeongsangnam-do 17) Jeju

**6. How is your child’s health?**

1) Very good

2) Good

3) Normal

4) Not good

5) Very bad

**7. Has your child been vaccinated over the past five years? (e.g., measles/spectrum/fungus (MMR), diphtheria/tide/whitening (DTP), Japanese encephalitis, flu, etc.)**

1) Yes 2) No 3) I do not know/ I do not remember

**8. Which of the following is the case for you(parents) regarding COVID-19 vaccination?**

1) I’ve completed the 3^rd^ vaccination.

2) I’ve completed the 2^nd^ vaccination.

3) I’ve completed the 1^st^ vaccination.

4) I’m not vaccinated.

**9. How likely do you think your child may get COVID-19?**

1) Extremely unlikely

2) Somewhat unlikely

3) Neutral

4) Somewhat likely

5) Extremely likely

**10. If your child gets COVID-19, how likely do you think that its effects are serious on his/her health?**

1) Not serious or severe at all

2) Not serious or severe

3) Neutral

4) Somewhat serious or severe

5) Extremely serious or severe

**11. Which of the following is close to the degree of confidence that you have the information you need for your children's COVID-19 vaccination?**

1) Not sure at all

2) Pretty sure

3) Sure it’s in the middle

4) Sure

5) Very sure

**<** **The following are data on COVID-19 infection and vaccines in children and adolescents in Korea. Please take the time to review this material before moving to the next questions.>**
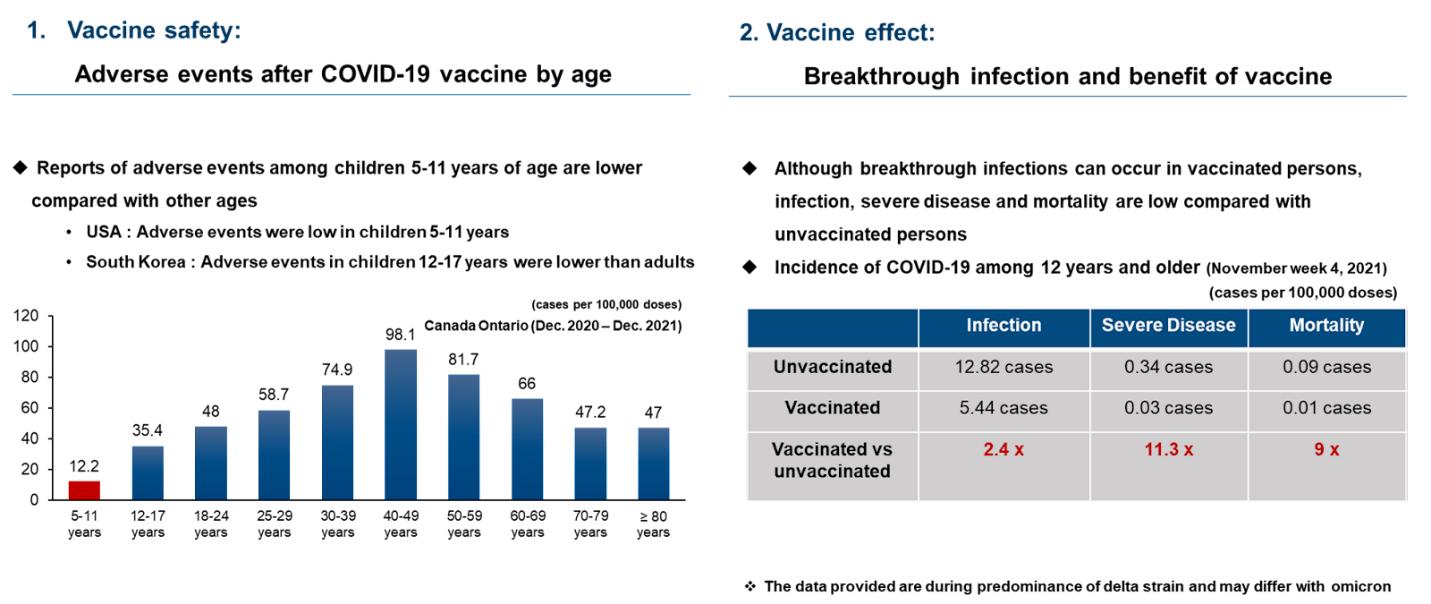


**12. Which of the following is your intention for your children’s COVID-19 vaccination?**

1) Willing to get vaccinated as soon as possible.

2) Willing to get vaccinated, but I want to see.

3) Don’t want to get vaccinated, but I want to see.

4) Not willing to get vaccinated and I’m not going to vaccinate my child.

5) Don’t know/ Not sure.

**13. Which of the following is close to your thoughts on the safety of COVID-19 vaccines?**

1) Very not safe

2) Not safe

3) Safe

4) Very safe

5) I do not know

**14. What do you think about the effectiveness of the COVID-19 vaccine to prevent infection and prevent severe deaths?**

1) Not effective at all

2) Not effective

3) Effective

4) Very effective

5) I do not know

**15. If you were to compare the benefits and risks of getting a COVID-19**

**vaccine for your child's health or daily life, which of the following do you think is closer?**

1) Risk is much greater than the benefit

2) Risk is greater than the benefit

3) Risks and benefits are half and half

4) Benefits are greater than the risks

5) Benefits are much greater than the risks

6) I do not know

**16. If you compare the risk of COVID-19 infection with the risk of getting vaccinated for the health and daily life of children aged 5-11, which of the following do you think is closer?**

1) For children of this age, the risk of COVID-19 infection is much greater than the risk of vaccination.

2) For children of this age, the risk of COVID-19 infection is greater than the risk of vaccination

3) The risk of COVID-19 infection in children of this age is the same as the risk of being vaccinated.

4) For children of this age, the risk of vaccination is greater than the risk of COVID-19 infection

5) For children of this age, the risk of vaccination is much greater than the risk of COVID-19 infection

6) I do not know

**17. If you recommend your child to be vaccinated, choose the two most important reasons.**

1) To prevent my child from being infected and to prevent serious infections and complications caused by infection

2) To prevent my child from spreading the infection to family or people around him/her.

3) To let my child get back to his/her pre-COVID-19 life by getting vaccinated

4) In order for our society to recover daily life such as economic recovery

5) To avoid the inconvenience of quarantine activities (body temperature measurement, meal partition, mask use, etc.)

6) Not applicable

**18. If you do not recommend your child to be vaccinated, choose the two most important reasons.**

1) Don't want to give my child an injection

2) To avoid my child experiencing adverse reactions or side effects after vaccination

3) To avoid disturbing my child's daily life and study, concerning about vaccination

4) don't think my child will get infected even if he/she doesn't get vaccinated.

5) The quarantine activities (body temperature measurement, meal partition, mask use, etc.) are enough, so don't want my child to get vaccinated.

6) Not applicable

**19. Which of the following is close to the level of parents' consent to the validity or reliability of the COVID-19 vaccine information currently provided or accessed?**

1) Strongly disagree

2) Disagree

3) Neither agree nor disagree

4) Agree

5) Strongly agree

**20. What is your opinion on COVID-19 vaccination?**

1) Vaccination is for myself

2) Vaccination is for the social community

3) Both

4) Neither

5) Don’t know

**21. Mark all relevant experiences related to COVID-19.**

1) Me/my family (parents, siblings, grandparents, etc.) have been infected

2) Me/my family (parents, siblings, grandparents, etc.) have been quarantined as a close contact

3) My child's school (or academy) teacher or friend has been infected

4) My child's school (or academy) teacher or friend has been quarantined as a close contact

5) Not applicable

**22. How safe do you think your child's school is from COVID-19?**

1) Not very safe

2) Not safe

3) Normal

4) Safe

5) Very safe

**The original Korean version of the survey can be found through the link.**

Vaccine profile informed group (VI group)

(<https://www.dropbox.com/s/t327vl614fpvf18/%EA%B5%90%EC%9C%A1%EB%B6%80%20%EC%BD%94%EB%A1%9C%EB%82%9819%20%EB%B0%B1%EC%8B%A0%20%EC%9D%B8%EC%8B%9D%20%EC%84%A4%EB%AC%B8%EC%A1%B0%EC%82%AC%20%28VIG%29.pdf?dl=0>)

Disease informed group (DI group)

(<https://www.dropbox.com/s/6m7iyh1iigd57yw/%EA%B5%90%EC%9C%A1%EB%B6%80%20%EC%BD%94%EB%A1%9C%EB%82%9819%20%EB%B0%B1%EC%8B%A0%20%EC%9D%B8%EC%8B%9D%20%EC%84%A4%EB%AC%B8%EC%A1%B0%EC%82%AC%20%28DIG%29.pdf?dl=0>)

Control group

(<https://www.dropbox.com/s/v4peagax0oeysat/%EA%B5%90%EC%9C%A1%EB%B6%80%20%EC%BD%94%EB%A1%9C%EB%82%9819%20%EB%B0%B1%EC%8B%A0%20%EC%9D%B8%EC%8B%9D%20%EC%84%A4%EB%AC%B8%EC%A1%B0%EC%82%AC%20%28Control%29.pdf?dl=0>)

1. **Additional results**

Table 2S shows the distribution of the participants in the survey in comparison to the total population of children grade 1 to grade 6. The total population data was retrieved from the Korea Education Statistics Service.

Table 2S. Representativeness of the participants in the survey in comparison with the total population of children of grade 1 to grade 6

|  | **Variable** |  | **Our survey**  (N = 359,110 ) | | **Total Population**  (N = 2,664,278) | | **Response rate** |
| --- | --- | --- | --- | --- | --- | --- | --- |
| **Gender of child** | Male |  | 184,648 | (51.4) | 1,366,211 | (51.3) | 13.5% |
|  | Female |  | 174,462 | (48.6) | 1,298,067 | (48.7) | 13.4% |
| **School grade** | Grade 1 |  | 11,251 | (3.1) | 431,222 | (16.2) | 2.6% |
|  | Grade 2 |  | 61,355 | (17.1) | 424,081 | (15.9) | 14.5% |
|  | Grade 3 |  | 62,106 | (17.3) | 424,454 | (15.9) | 14.6% |
|  | Grade 4 |  | 71,617 | (19.9) | 470,779 | (17.7) | 15.2% |
|  | Grade 5 |  | 75,188 | (20.9) | 458,085 | (17.2) | 16.4% |
|  | Grade 6 |  | 77,593 | (21.6) | 455,657 | (17.1) | 17.0% |
| **Region** | Seoul, Incheon |  | 84,253 | (23.5) | 549,471 | (20.6) | 15.3% |
|  | Gyeonggi |  | 83,804 | (23.3) | 767,346 | (28.8) | 10.9% |
|  | Daejeon, Sejong, Chungcheong |  | 34,801 | (9.7) | 311,245 | (11.7) | 11.2% |
|  | Gwangju, Jeolla |  | 34,726 | (9.7) | 265,457 | (10.0) | 13.1% |
|  | Busan, Ulsan, Gyeongsangnam-do |  | 67,560 | (18.8) | 408,731 | (15.3) | 16.5% |
|  | Daegu, Gyeongsangbuk-do |  | 38,330 | (10.7) | 248,870 | (9.3) | 15.4% |
|  | Gangwon, Jeju |  | 15,636 | (4.4) | 113,158 | (4.2) | 13.8% |

Table 3S shows the results of the balance check. Column (2), (3) display the difference in means between the treatment group and the control group. Column (4) displays the p-value of the F-test under the null hypothesis that the means across the three groups are the same. Standard errors in parenthesis are clustered at the area level.

Table 3S. Balance check

|  | (1) | (2) | (3) | (4) |
| --- | --- | --- | --- | --- |
| Variables | Control group | VI Group | DI Group | F-value |
|  | (Std.errors) | (Std.errors) | (Std.errors) | (p-value) |
| Gender | 0.514 | 0.00136 | -0.00144 | 1.023 |
|  | (0.00148) | (0.00196) | (0.00140) | (0.382) |
| Grade1 | 0.0317 | -0.00134 | 0.000148 | 0.206 |
|  | (0.00479) | (0.00222) | (0.00213) | (0.816) |
| Grade2 | 0.170 | 0.000969 | 0.000798 | 0.107 |
|  | (0.00199) | (0.00220) | (0.00265) | (0.899) |
| Grade3 | 0.172 | 0.00233 | 0.00129 | 0.571 |
|  | (0.00175) | (0.00218) | (0.00194) | (0.576) |
| Grade4 | 0.200 | -0.00129 | -0.00111 | 0.535 |
|  | (0.00153) | (0.00144) | (0.00137) | (0.596) |
| Grade5 | 0.211 | -0.00222 | -0.00201 | 0.804 |
|  | (0.00197) | (0.00242) | (0.00159) | (0.465) |
| Grade6 | 0.215 | 0.00154 | 0.000887 | 0.276 |
|  | (0.00456) | (0.00215) | (0.00149) | (0.762) |
| 1, 2 children | 0.863 | -0.000789 | 0.00265 | 1.228 |
|  | (0.00930) | (0.00228) | (0.00183) | (0.319) |
| 3+ children | 0.137 | 0.000789 | -0.00265 | 1.228 |
|  | (0.00930) | (0.00228) | (0.00183) | (0.319) |

|  | (1) | (2) | (3) | (4) |
| --- | --- | --- | --- | --- |
| Variables | Control group | VI Group | DI Group | F-value |
|  | (Std.errors) | (Std.errors) | (Std.errors) | (p-value) |
| Seoul, Incheon | 0.231 | -0.0169 | 0.0242 | 1.455 |
|  | (0.159) | (0.0250) | (0.0237) | (0.263) |
| Gyeonggi | 0.252 | -0.0450 | -0.0123 | 1.243 |
|  | (0.204) | (0.0285) | (0.0133) | (0.315) |
| Daejeon, Sejong, Chungcheong | 0.0964 | 0.00682 | -0.00491 | 0.981 |
|  | (0.0617) | (0.0129) | (0.00762) | (0.396) |
| Gwangju, Jeolla | 0.106 | -0.00986 | -0.0180 | 0.836 |
|  | (0.0686) | (0.0143) | (0.0144) | (0.452) |
| Busan, Ulsan,  Gyeongsangnam-do | 0.169 | 0.0480 | 0.00932 | 2.316 |
|  | (0.114) | (0.0276) | (0.0283) | (0.131) |
| Daegu, Gyeongsangbuk-do | 0.0997 | 0.0217 | -7.57e-05 | 0.812 |
|  | (0.0744) | (0.0171) | (0.00631) | (0.461) |
| Gangwon, Jeju | 0.0445 | -0.00480 | 0.00176 | 0.268 |
|  | (0.0363) | (0.00711) | (0.00313) | (0.768) |
| 3^rd^ shot | 0.489 | -0.00326 | -0.000932 | 0.296 |
|  | (0.0101) | (0.00447) | (0.00469) | (0.748) |
| 2^nd^ shot | 0.395 | 0.00297 | 0.000822 | 0.311 |
|  | (0.0105) | (0.00409) | (0.00421) | (0.737) |
| 1^st^ shot & none | 0.117 | 0.000294 | 0.000110 | 0.0221 |
|  | (0.00315) | (0.00149) | (0.00170) | (0.978) |

| Table 4-1S. Average response rates on parental intention of COVID-19 vaccination for their children and parental perceptions across groups | | | | | | | | | | |
| --- | --- | --- | --- | --- | --- | --- | --- | --- | --- | --- |
|  |  |  | Control group | |  | VI Group | |  | DI Group | |
| **Outcome** | |  | Mean response rate  (95% CI) | No. obs |  | Mean response rate  (95% CI) | No. obs |  | Mean response rate  (95% CI) | No. obs |
| **Intention to vaccinate children** | |  |  |  |  |  |  |  |  |  |
| Strong | |  | 0.0650 | 7,379 |  | 0.0730 | 8,561 |  | 0.0712 | 9,139 |
|  |  |  | (0.0580 - 0.0721) |  |  | (0.0651 - 0.0809) |  |  | (0.0644 - 0.0780) |  |
| Strong or moderate | |  | 0.354 | 40,118 |  | 0.368 | 43,181 |  | 0.362 | 46,503 |
|  |  |  | (0.330 - 0.377) |  |  | (0.343 - 0.394) |  |  | (0.340 - 0.384) |  |
| No | |  | 0.610 | 69,204 |  | 0.598 | 70,174 |  | 0.606 | 77,859 |
|  |  |  | (0.585 - 0.635) |  |  | (0.572 - 0.625) |  |  | (0.583 - 0.630) |  |
|  | | | | | | | | | | |

|  |  |  | Control group | |  | VI Group | |  | DI Group | |
| --- | --- | --- | --- | --- | --- | --- | --- | --- | --- | --- |
| Outcome | |  | Mean response rate  (95% CI) | No. obs |  | Mean response rate  (95% CI) | No. obs |  | Mean response rate  (95% CI) | No. obs |
| **Perception on Risk-Benefit** | |  |  |  |  |  |  |  |  |  |
| Benefit > Risk | |  | 0.119 | 13,469 |  | 0.131 | 15,313 |  | 0.124 | 15,873 |
|  |  |  | (0.110 - 0.127) |  |  | (0.119 - 0.142) |  |  | (0.114 - 0.134) |  |
| Risk > Benefit | |  | 0.517 | 58,641 |  | 0.478 | 56,090 |  | 0.498 | 63,915 |
|  |  |  | (0.496 - 0.538) |  |  | (0.456 - 0.500) |  |  | (0.477 - 0.518) |  |
| **Perception on risk comparison of COVID-19 infection and vaccine** | |  |  |  |  |  |  |  |  |  |
| Infection risk > vaccine risk | |  | 0.149 | 16,938 |  | 0.165 | 19,349 |  | 0.162 | 20,809 |
|  |  |  | (0.142 - 0.157) |  |  | (0.157 - 0.173) |  |  | (0.155 - 0.169) |  |
| vaccine risk > infection risk | |  | 0.521 | 59,103 |  | 0.485 | 56,913 |  | 0.496 | 63,630 |
|  |  |  | (0.495 - 0.547) |  |  | (0.459 - 0.511) |  |  | (0.471 - 0.520) |  |
| **Self-reported trust in COVID-19 vaccine information** | |  |  |  |  |  |  |  |  |  |
| Yes | |  | 0.203 | 23,021 |  | 0.211 | 24,754 |  | 0.206 | 26,490 |
|  |  |  | (0.190 - 0.216) |  |  | (0.197 - 0.226) |  |  | (0.193 - 0.220) |  |
|  | | | | | | | | | | |

| Table 4-2S. Conditional average treatment effects | | | | | | |  |  | | |
| --- | --- | --- | --- | --- | --- | --- | --- | --- | --- | --- |
|  |  | |  | VI Group (Conditional) | | |  | DI Group (Conditional) | | |
| **Outcome** | | |  | DRR  (95% CI) | p-value | Normalized DRR |  | DRR  (95% CI) | p-value | Normalized DRR |
| **Intention to vaccinate children** | | |  |  |  |  |  |  |  |  |
| Strong | | |  | 0.00847 | <.001 | 0.130 |  | 0.00669 | <.001 | 0.103 |
|  | |  |  | (0.00653 - 0.0104) |  |  |  | (0.00372 - 0.00965) |  |  |
| Strong or moderate | | |  | 0.0162 | <.001 | 0.046 |  | 0.0110 | .002 | 0.031 |
|  | |  |  | (0.0123 - 0.0201) |  |  |  | (0.00458 - 0.0174) |  |  |
| No | | |  | -0.0134 | <.001 | -0.022 |  | -0.00623 | .074 | -0.010 |
|  | |  |  | (-0.0182 - -0.00855) |  |  |  | (-0.0131 - 0.000673) |  |  |
|  | | | | | | | | | | |

|  |  |  | VI Group (Conditional) | | |  | DI Group (Conditional) | | |
| --- | --- | --- | --- | --- | --- | --- | --- | --- | --- |
| Outcome | |  | DRR  (95% CI) | p-value | Normalized DRR |  | DRR  (95% CI) | p-value | Normalized DRR |
| **Perception on Risk-Benefit** | |  |  |  |  |  |  |  |  |
| Benefit > Risk | |  | 0.0133 | <.001 | 0.112 |  | 0.00523 | .009 | 0.044 |
|  |  |  | (0.0101 - 0.0165) |  |  |  | (0.00151 - 0.00894) |  |  |
| Risk > Benefit | |  | -0.0399 | <.001 | -0.077 |  | -0.0215 | <.001 | -0.042 |
|  |  |  | (-0.0441 - -0.0357) |  |  |  | (-0.0281 - -0.0148) |  |  |
| **Perception on risk comparison of COVID-19**  **infection and vaccine** | |  |  |  |  |  |  |  |  |
| Infection risk > vaccine risk | |  | 0.0167 | <.001 | 0.112 |  | 0.0136 | <.001 | 0.091 |
|  |  |  | (0.0120 - 0.0213) |  |  |  | (0.00937 - 0.0179) |  |  |
| vaccine risk > infection risk | |  | -0.0367 | <.001 | -0.070 |  | -0.0285 | <.001 | -0.055 |
|  |  |  | (-0.0421 - -0.0312) |  |  |  | (-0.0349 - -0.0221) |  |  |
| **Self-reported trust in COVID-19 vaccine**  **information** | |  |  |  |  |  |  |  |  |
| Yes | |  | 0.00963 | .002 | 0.047 |  | 0.00422 | .067 | 0.021 |
|  | | | | | | | | | |

Table 4-3S. Unconditional average treatment effects

The normalized difference of mean response rates between treatment group and control group (normalized DRR) to the mean response rate in the control group is also reported. The regression reported here does not contain any control variable.

| Outcome | |  | VI Group (Unconditional) | | |  | DI Group (Unconditional) | | |
| --- | --- | --- | --- | --- | --- | --- | --- | --- | --- |
|  |  |  | DRR (95% CI) | p-value | Normalized DRR |  | DRR (95% CI) | p-value | Normalized DRR |
| **Intention to vaccinate children** | |  |  |  |  |  |  |  |  |
| Strong | |  | 0.00796 | <.001 | 0.122 |  | 0.00614 | .002 | 0.094 |
|  |  |  | (0.00490 - 0.0110) |  |  |  | (0.00261 - 0.00966) |  |  |
| Strong or moderate | |  | 0.0146 | .017 | 0.041 |  | 0.00857 | .44 | 0.024 |
|  |  |  | (0.00646 - 0.0228) |  |  |  | (-0.000499 - 0.0176) |  |  |
| No | |  | -0.0116 | <.001 | -0.019 |  | -0.00360 | .07 | -0.006 |
|  |  |  | (-0.0207 - -0.00239) |  |  |  | (-0.0132 - 0.00604) |  |  |

| Outcome | |  | VI Group (Unconditional) | | |  | DI Group (Unconditional) | | |
| --- | --- | --- | --- | --- | --- | --- | --- | --- | --- |
|  |  |  | DRR (95% CI) | p-value | Normalized DRR |  | DRR (95% CI) | p-value | Normalized DRR |
| **Perception on Risk-Benefit** | |  |  |  |  |  |  |  |  |
| Benefit > Risk | |  | 0.0119 | <.001 | 0.100 |  | 0.00490 | .036 | 0.041 |
|  |  |  | (0.00711 - 0.0166) |  |  |  | (0.000376 - 0.00943) |  |  |
| Risk > Benefit | |  | -0.0386 | <.001 | -0.075 |  | -0.0191 | <.001 | -0.037 |
|  |  |  | (-0.0453 - -0.0318) |  |  |  | (-0.0278 - -0.0104) |  |  |
| **Perception on risk comparison of COVID-19 infection and vaccine** | |  |  |  |  |  |  |  |  |
| Infection risk > vaccine risk | |  | 0.0157 | <.001 | 0.105 |  | 0.0128 | <.001 | 0.086 |
|  |  |  | (0.0106 - 0.0208) |  |  |  | (0.00796 - 0.0176) |  |  |
| vaccine risk > infection risk | |  | -0.0356 | <.001 | -0.068 |  | -0.0254 | <.001 | -0.049 |
|  |  |  | (-0.0434 - -0.0278) |  |  |  | (-0.0333 - -0.0175) |  |  |
| **Self-reported trust in COVID-19 vaccine information** | |  |  |  |  |  |  |  |  |
| Yes | |  | 0.00818 | .01 | 0.040 |  | 0.00340 | .089 | 0.017 |
|  |  |  | (0.00227 - 0.0141) |  |  |  | (-0.000577 - 0.00737) |  |  |

Table 5S. Average treatment effects on parental intentions to vaccinate their children

This table shows the average treatment effect on each of vaccination intentions (strong, strong or moderate, and hesitant.)

Columns (1), (2), and (3) display the results without controlling any baseline covariates and Columns (4), (5), and (6) display the results with the full set of controls including demographic characteristics. Standard errors in parentheses are clustered at the area level. (* p<0.10 ** p<0.05 *** p<0.01.)

| ATE | (1) | (2) | (3) | (4) | (5) | (6) |
| --- | --- | --- | --- | --- | --- | --- |
| VI Group | 0.00796*** | 0.0146*** | -0.0116** | 0.00847*** | 0.0162*** | -0.0134*** |
|  | (0.00145) | (0.00385) | (0.00433) | (0.000916) | (0.00184) | (0.00228) |
| DI Group | 0.00614*** | 0.00857* | -0.00360 | 0.00669*** | 0.0110*** | -0.00623* |
|  | (0.00166) | (0.00428) | (0.00455) | (0.00140) | (0.00302) | (0.00326) |
| Obs. | 359,110 | 359,110 | 359,110 | 359,110 | 359,110 | 359,110 |
| R-squared | 0.000 | 0.000 | 0.000 | 0.092 | 0.192 | 0.186 |
| Dep. Var. mean of control group | 0.065 | 0.354 | 0.610 | 0.065 | 0.354 | 0.610 |
| Vaccination intention | Strong | Moderate | Hesitant | Strong | Moderate | Hesitant |
| Demographic info. | No | No | No | Yes | Yes | Yes |
| Other controls | No | No | No | Yes | Yes | Yes |
|  | | | | | | |

Table 6S. Heterogeneous treatment effects on intention to vaccinate.

This table display the heterogeneous treatment effects on strong or moderate vaccination intention. (* p<0.10, ** p<0.05, *** p<0.01.)

| Variables | Gender | | Number | | Health status of a child | | |
| --- | --- | --- | --- | --- | --- | --- | --- |
|  | Female | male | 1,2 children | 3+ children | Healthy | Moderate | Unhealthy |
| VI Group | 0.0164*** | 0.0161*** | 0.0167*** | 0.0130*** | 0.0158*** | 0.0166*** | 0.0410*** |
|  | (0.00259) | (0.00252) | (0.00194) | (0.00500) | (0.00193) | (0.00538) | (0.0152) |
| DI Group | 0.0122*** | 0.00979*** | 0.0107*** | 0.0134*** | 0.0110*** | 0.00901* | 0.0313** |
|  | (0.00252) | (0.00246) | (0.00188) | (0.00492) | (0.00188) | (0.00523) | (0.0150) |
| Observation | 174462 | 184648 | 310168 | 48942 | 318370 | 37086 | 3654 |
| R-squared | 0.186 | 0.192 | 0.185 | 0.197 | 0.189 | 0.163 | 0.142 |
| Dep. Var. mean of control group | 0.347 | 0.360 | 0.342 | 0.427 | 0.364 | 0.281 | 0.177 |

| Variables | Grade | | | | | |
| --- | --- | --- | --- | --- | --- | --- |
|  | Grade1 | Grade2 | Grade3 | Grade4 | Grade5 | Grade6 |
| VI Group | -0.0113 | 0.00998** | 0.0119*** | 0.0169*** | 0.0215*** | 0.0225*** |
|  | (0.00990) | (0.00412) | (0.00417) | (0.00404) | (0.00406) | (0.00406) |
| DI Group | -0.0194** | 0.00905** | 0.0113*** | 0.00993** | 0.0110*** | 0.0170*** |
|  | (0.00958) | (0.00403) | (0.00408) | (0.00393) | (0.00395) | (0.00396) |
| Observation | 11251 | 61355 | 62106 | 71617 | 75188 | 77593 |
| R-squared | 0.149 | 0.140 | 0.160 | 0.163 | 0.174 | 0.188 |
| Dep. Var. mean of control group | 0.291 | 0.259 | 0.285 | 0.328 | 0.391 | 0.480 |

| Variables | Past vaccination of a child | | | Parent's Covid-19 vaccination | | | Chance of Covid-19 infection (child) | | |
| --- | --- | --- | --- | --- | --- | --- | --- | --- | --- |
|  | Yes | No | Unknown | 3rd shot | 2nd shot | 1st shot & none | Unlikely | Neutral | Likely |
| VI Group | 0.0155*** | 0.0179** | 0.0229*** | 0.0198*** | 0.0129*** | 0.0116*** | 0.0150*** | 0.0156*** | 0.0193*** |
|  | (0.00192) | (0.00761) | (0.00720) | (0.00279) | (0.00266) | (0.00447) | (0.00361) | (0.00239) | (0.00424) |
| DI Group | 0.0103*** | 0.00441 | 0.0265*** | 0.0137*** | 0.00933*** | 0.00630 | 0.00630* | 0.00989*** | 0.0202*** |
|  | (0.00188) | (0.00731) | (0.00709) | (0.00272) | (0.00259) | (0.00434) | (0.00351) | (0.00233) | (0.00412) |
| Observation | 316381 | 20617 | 22112 | 174960 | 142249 | 41901 | 84901 | 205222 | 68987 |
| R-squared | 0.189 | 0.194 | 0.180 | 0.132 | 0.089 | 0.163 | 0.199 | 0.175 | 0.197 |
| Dep. Var. mean of control group | 0.355 | 0.358 | 0.327 | 0.498 | 0.222 | 0.194 | 0.321 | 0.341 | 0.429 |

| Variables | Health risk of Covid-19 infection (child) | | | Confidence on Covid-19 vaccine information | | |
| --- | --- | --- | --- | --- | --- | --- |
|  | Mild | Moderate | Severe | Not at all | Somewhat | Completely |
| VI Group | 0.00811* | 0.0190*** | 0.0160*** | 0.0202*** | 0.0165*** | 0.00674 |
|  | (0.00422) | (0.00285) | (0.00275) | (0.00285) | (0.00249) | (0.00456) |
| DI Group | 0.00608 | 0.0147*** | 0.00913*** | 0.0150*** | 0.0109*** | 0.00514 |
|  | (0.00408) | (0.00278) | (0.00269) | (0.00274) | (0.00244) | (0.00443) |
| Observation | 50799 | 144802 | 163509 | 91629 | 217151 | 50330 |
| R-squared | 0.200 | 0.175 | 0.181 | 0.058 | 0.125 | 0.298 |
| Dep. Var. mean of control group | 0.232 | 0.339 | 0.404 | 0.137 | 0.440 | 0.378 |
|  | | | | | | |

Table 7S. Response time

Table 7S displays the treatment effects on response time in seconds. Column (1) reports the results without controlling any baseline covariates and Column (2) with the full set of controls including demographic characteristics. Compared to the control group in which the average response time is 415 seconds, we observe subjects taking 41 seconds more in the VI Group and 43 seconds more in the DI Group. These results remain robust with the full set of controls. * p<0.10, ** p<0.05, *** p<0.01.

| Response time | (1) | (2) |
| --- | --- | --- |
| VI Group | 40.84*** | 41.36*** |
|  | (5.612) | (5.464) |
| DI Group | 43.35*** | 44.28*** |
|  | (8.516) | (8.825) |
| Obs. | 359,110 | 359,110 |
| R-squared | 0.000 | 0.001 |
| Dep. Var. mean of  control group | 415.1 | 415.1 |
| Demographic info. | No | Yes |
| Other controls | No | Yes |
|  | | |
